# Kaempferol From *Penthorum chinense* Pursh Attenuates Hepatic Ischemia/Reperfusion Injury by Suppressing Oxidative Stress and Inflammation Through Activation of the Nrf2/HO-1 Signaling Pathway

**DOI:** 10.3389/fphar.2022.857015

**Published:** 2022-04-01

**Authors:** Yifan Chen, Tongxi Li, Peng Tan, Hao Shi, Yonglang Cheng, Tianying Cai, Junjie Bai, Yichao Du, Wenguang Fu

**Affiliations:** ^1^ Department of General Surgery (Hepatopancreatobiliary Surgery), The Affiliated Hospital of Southwest Medical University, Luzhou, China; ^2^ Academician (Expert) Workstation of Sichuan Province, The Affiliated Hospital of Southwest Medical University, Luzhou, China

**Keywords:** kaempferol, ischemia/reperfusion, oxidative stress, inflammation, Nrf2/HO-1

## Abstract

The purpose of this study is to investigate the protective effect of kaempferol (KAE), the main active monomer from *Penthorum chinense* Pursh, on hepatic ischemia/reperfusion injury (HI/RI) and its specific mechanism. HI/RI is a common complication closely related to the prognosis of liver surgery, and effective prevention and treatment methods are still unavailable. Ischemia/reperfusion (I/R) injury is caused by tissue damage during ischemia and sustained oxidative stress and inflammation during reperfusion. *Penthorum chinense* Pursh is a traditional Chinese medicine widely used to treat liver disease since ancient times. Kaempferol (KAE), a highly purified flavonoid active monomer isolated and extracted from *Penthorum chinense* Pursh, was investigated for its protective effect on HI/RI. Our study indicates that KAE pretreatment alleviated I/R-induced transaminase elevation and pathological changes. Further analysis revealed that KAE pretreatment attenuates I/R-induced oxidative stress (as measured by the content of MDA, SOD and GSH) *in vivo* and reduces hypoxia/reoxygenation (H/R) -induced reactive oxygen species (ROS) generation *in vitro*. Meanwhile, KAE inhibits activation of NF-κB/p65 and reduces the release of pro-inflammatory factors (TNF-α and IL-6) to protect the liver from I/R-induced inflammation. Nuclear erythroid 2-related factor 2 (Nrf2) is a crucial cytoprotection regulator because it induces anti-inflammatory, antioxidant, and cytoprotective genes. Therefore, we analyzed the protein levels of Nrf2 and its downstream heme oxygenase-1 (HO-1) in the liver of mice and hepatocytes of humankind, respectively, and discovered that KAE pretreatment activates the Nrf2/HO-1 signaling pathway. In summary, this study confirmed the hepatoprotective effect of KAE on HI/RI, which inhibits oxidative stress and inflammation by activating the Nrf2/HO-1 signaling pathway.

## Introduction

HI/RI is a pathological state characterized by initial restriction of blood flow to the organ followed by restoration of perfusion and concomitant reoxygenation. However, blood flow restoration and reoxygenation are frequently associated with worsening tissue damage and a severe inflammatory response (Eltzschig and Eckle, 2011). HI/RI is a severe and unavoidable complication of certain liver surgeries, particularly partial hepatectomy and liver transplantation. It can result in delayed recovery of liver function and nonfunctioning of the transplanted liver following surgery, compromising the prognosis of liver surgery (Jochmans et al., 2017). HI/RI is a biphasic pathophysiological process that consists of two phases; the ischemic phase and the reperfusion phase. During ischemia, activated Kupffer cells release ROS, TNF-α, and IL-1β, leading to subsequent leukocyte recruitment, hepatocyte death, and endothelial injury (Ju and Tacke, 2016; Abu-Amara et al., 2010). Meanwhile, reoxygenation during the reperfusion period will lead to acute ROS generation, and the rapid accumulation of ROS directly causes tissue damage and impairs mitochondrial function and antioxidant systems, further exacerbating the deleterious effects of ROS, leading to sterile inflammation, apoptosis, and organ failure (Elias-Miró et al., 2013).

Natural product-based drugs have been regarded as a novel therapeutic strategy for preventing and treating certain diseases in recent years. Previous studies have shown that herbal active monomers have tremendous therapeutic potential, with pharmacological effects, including anti-inflammatory, antioxidant, and anti-apoptotic (Subramanya et al., 2018). *Penthorum chinense* Pursh (also known as Ganhuangcao in traditional Chinese medicine) is a medicinal and edible herb native to Miao nationality in China that grows primarily in southwest China (especially in Gulin County, Luzhou, Sichuan). *Penthorum chinense* Pursh has been used to treat liver diseases and alleviate liver injury in acute and chronic hepatitis, liver fibrosis, and non-alcoholic fatty liver disease for thousands of years (Wang et al., 2015). The main active ingredients of *Penthorum chinense* Pursh include flavonoids, organic acids, sterols, lignans, and volatile oils (Guo et al., 2015).

**FIGURE 1:**
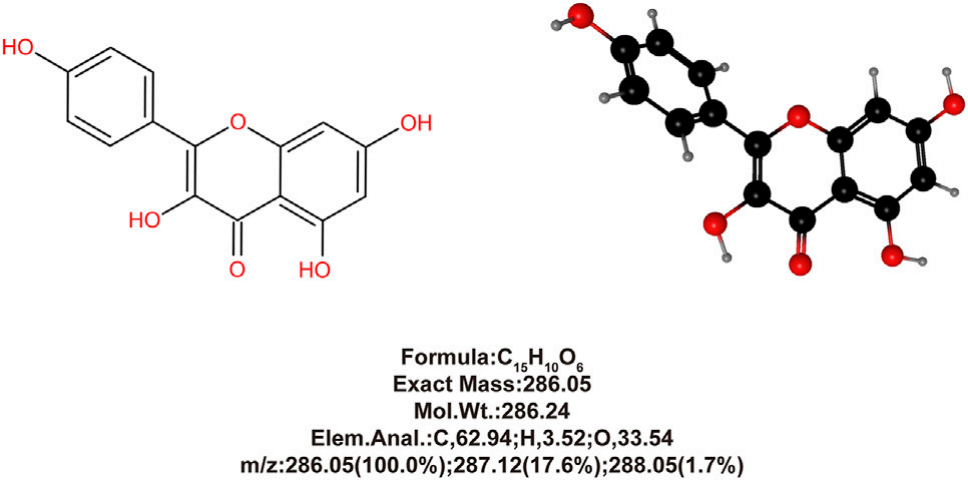
Chemical and 3D structure of KAE and chemical properties.

In our previous study (Du et al., 2020), we isolated and extracted high purity KAE (purity >98%, based on High-performance liquid chromatography (HPLC) analysis) (Figure 1) from *Penthorum chinense* Pursh and demonstrated its significant anti-inflammatory, antioxidant, and anti-apoptotic effects using the acetaminophen (N-acetyl-p-aminophenol, APAP)-induced hepatotoxicity mice model. Furthermore, Rabha et al. (Rabha et al., 2018) demonstrated that in a mice model of sepsis-induced acute lung injury, KAE pretreatment reduced the levels of cytokines IL-6, IL-1β, and TNF-α in plasma and lung tissue and increases the antioxidant products, SOD and GSH, to attenuate inflammation and oxidative stress.

Current studies widely indicate that oxidative stress and inflammation are critical mechanisms for the occurrence and progression of HI/RI, which is induced by the release of inflammatory factors (primarily TNF-α and IL-6, etc.) and the accumulation of ROS. Therefore, inhibiting oxidative stress and inflammation following liver surgery is a feasible therapeutic strategy for alleviating HI/RI (Pizzino et al., 2017; Nace et al., 2013). Based on the above background, this study examined the protective effect of KAE on I/R injury using the HI/RI model of mice and the H/R model of hepatocytes, and investigated the specific mechanism of the hepatoprotective effects of KAE.

## Materials and Methods

### KAE Extraction and Isolation

The method for isolating and extracting KAE from *Penthorum chinense* Pursh is described in our previous study (Du et al., 2020), and the purity was confirmed to be >98% based on HPLC analysis (Figure 1).

### Animals and Groups

We purchased 48 male C57BL/6 mice (8–10 weeks old, weighing 18–22 g) from Hua Fukang Bioscience (Beijing, China) and housed them under controlled light (12-h light/dark cycle) and temperature (22 ± 2°C) conditions with free access to food and water. After 1 week of adaptive feeding, the mice were randomly divided into the following groups (eight mice each): 1) Sham group (Sham); 2) KAE 60 mg/kg group (KAE60); 3) HI/RI group (HI/RI); 4) HI/RI + KAE 15 mg/kg (HI/RI + KAE15); 5) HI/RI + KAE 30 mg/kg (HI/RI + KAE30); 6) HI/RI + KAE 60 mg/kg (HI/RI + KAE60).

Mice in groups 2), 4), 5), and 6) were administered the above dose of KAE by gavage for 7 days, and HI/RI model was performed, with samples collected on day 8. All animal experiments in this study were reviewed and approved by the Animal Care and Use Committee and Ethics Committee of Southwest Medical University.

### Mice HI/RI Model

According to Yuta Abe’s method, nonlethal segmental (70%) liver ischemia was established. Briefly, all mice were anesthetized with sodium pentobarbital (40 mg/kg, i.p) before dissecting their abdomens along the midline. Then, using an atraumatic microvascular clamp, nonlethal segmental (70%) liver ischemia was induced by occlusion of the hepatic artery and portal vein of the left and median lobes. After 60 min of segmental liver ischemia, the clamps were removed to initiate liver reperfusion. The mice were sacrificed with overdose sodium pentobarbital (90 mg/kg, i. p) after 6 h of reperfusion, and plasma and liver tissues were collected for analysis. Additionally, the mice belonging to the Sham group underwent a midline laparotomy incision without microvascular clamp placement.

### Cell Culture and H/R Model of Hepatocytes

Normal human hepatocytes QSG-7701 were purchased from Beyotime Biotechnology (Shanghai, China) and cultured in Dulbecco’s modified Eagle’s medium (DMEM; Gibco; Thermo Fisher Scientific, United States) supplemented with 10% fetal bovine serum (Biological Industries, Beit Haemek, Israel) and 1% penicillin-streptomycin (Solarbio, Beijing, China) in a humidified incubator (Thermo Fisher Scientific, United States; 37°C, 5% CO_2_). The H/R model of hepatocytes was established with some modifications to our previous study (Du et al., 2019). Briefly, experimental group hepatocytes were pretreated with various concentrations of KAE for 24 h before H/R procedure, while the control group was treated with the same volume of the KAE vehicle (DMSO; Solarbio, Beijing China) and maintained in the incubator. After that, all hepatocytes were washed twice with warm PBS and replaced with glucose-free and serum-free DMEM (Balanced with 1% O_2_, 5% CO_2_, and 94% N_2_; Procell, Wuhan, China) 1 h before the hypoxia period. Experimental group cells were then cultured under hypoxic conditions (37°C, 1% O_2_, 5% CO_2,_ and balanced N_2_) in an InvivO_2_ 400 hypoxic workstation (Baker Ruskinn, United Kingdom) for 6 h. Then, both groups of hepatocytes were replaced with fresh warm DMEM in the incubator (37°C, 5% CO_2_) for a 4-h reoxygenation period.

### Serum Aminotransferase Analyses

Mice serum samples were obtained by centrifuging (4°C, 5,000 rpm, 5 min) blood. An automatic biochemical analyzer (ADVIA 2400 Chemistry System, Siemens, Germany) was used to determine the serum activities of ALT and AST in mice.

### Determination of Hepatic MDA, SOD and GSH Content

The supernatant was collected after the homogenization of the mice liver, and the content of MDA, SOD and GSH were measured according to the kit manufacturer’s instructions (Beyotime, Shanghai, China).

### Hematoxylin-Eosin and Immunohistochemistry Staining

Liver samples from each group of mice were collected immediately after the I/R procedure and fixed in a 4% paraformaldehyde solution for 24 h, followed by dehydration with gradient ethanol, paraffin embedding, and sectioning for H&E and IHC staining of HO-1 (1:500, Proteintech, Wuhan, China).

### Terminal Deoxynucleotidyl Transferase-Mediated dUTP-Biotin Nick End Labeling Apoptosis Assay

TUNEL apoptosis assay for mice liver tissue sections was performed according to the kit manufacturer’s instructions (Servicebio, Wuhan, China).

### Cell Viability Assay

According to the manufacturer’s instructions, cell viability was determined using the cell counting kit 8 (CCK-8; Beyotime, Shanghai, China). Briefly, hepatocytes (3 × 10^4^/well) were inoculated in a 96-well plate, then 10 μl CCK-8 solution was added and cultured routinely. After 2 h, absorbance at 450 nm was measured using a Cytation5 Imaging Reader (BioTek, United States), and cell viability was calculated. Duplicate wells were used in the respective groups and repeated four times.

### Detection of ROS Generation

For cellular ROS determination and fluorescence analysis, we loaded a dichlorofluorescein-diacetate (DCFH-DA) fluorescent probe according to the manufacturer’s instructions (Beyotime, Shanghai, China). The area scan function (3 × 3 reads/well; excitation/emission = 488/525 nm) of the Gen5 software (Vision.3.08; Biotek, United States) was used to calculate the average fluorescence intensity of 6-well plates, and subsequent fluorescence images were captured using a fluorescence microscope (IX73; Olympus, Tokyo, Japan).

### Western Blotting

According to the manufacturer’s instructions, RIPA lysis buffer (Beyotime, Shanghai, China) was used to extract total protein from liver tissue and hepatocytes. Western blotting was performed as previously described (Lei et al., 2016). The membranes were incubated with primary antibodies against NF-κB/p65 (1:1,000; Proteintech, United States), phospho-NF-κB/p65 (p-p65; 1:1,000; Zen Bioscience, China), TNF-α (1:1,000; Proteintech; United States), IL-6 (1:1,000; Proteintech, United States), IL-10 (1:1,000; Wanleibio, China), Bax (1:2000; Proteintech, United States), Bcl-2 (1:1,000; Proteintech, United States), Nrf2 (1:1,000; Proteintech, United States), HO-1 (1:1,000; Proteintech, United States), β-actin (1:5,000; Proteintech, United States) and then incubated with horseradish peroxidase (HRP)-conjugated secondary antibodies (1:5,000; Proteintech, United States). The relative protein expression was analyzed using ImageJ software (NIH, Maryland, United States).

### Statistical Analysis

All data analyses were performed using GraphPad Prism v.8.0 (GraphPad Software, San Diego, United States) and presented as mean ± standard deviation. All data were compared using one-way ANOVA and *t*-test, and a *p*-value less than 0.05 (*p* < 0.05) was considered to represent statistically significant results.

## Results

### KAE Mitigates Liver Injury in Mice HI/RI Model

**FIGURE 2:**
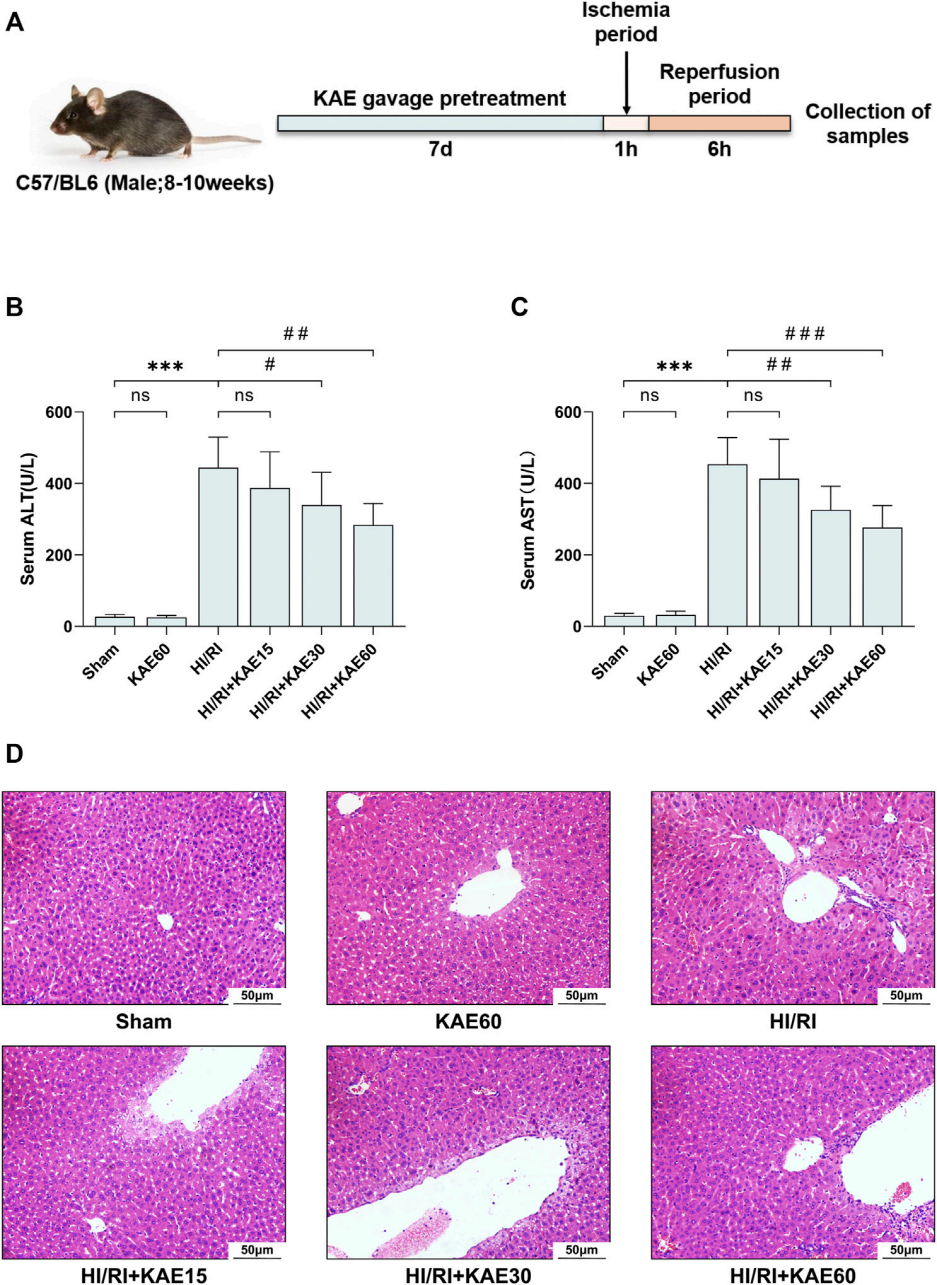
KAE mitigates liver injury in mice HI/RI model. **(A)** KAE administration and HIRI model establishment in mice (*n* = 8/group); Effect of KAE pretreatment on serum of ALT **(B)** and AST **(C)**; **(D)** Pathological evaluation of liver tissue specimens by H&E staining (original magnification ×200). Data were expressed as mean ± standard deviation (SD) values. *p < 0.05, ∗∗*p* < 0.01, and ∗∗∗*p* < 0.001 versus the sham group; ^#^ *p* < 0.05, ^##^ *p* < 0.01, and ^###^ *p* < 0.001 versus the HI/RI group; NS: no significance.

In this study, mice were pretreated with different doses of KAE gavage (15, 30, and 60 mg/kg) for 7 days before establishing HI/RI models, as previously described (Figure 2A). We first evaluated the effect of KAE on the liver of mice and observed no significant difference in liver function (*p* > 0.05) (Figures 2B,C) and tissue structure (Figure 2D) between the KAE60 and Sham groups of mice, confirming that the dose of KAE gavage was not significantly toxic to mice. While KAE pretreatment dose-dependently reduced I/R-induced transaminase elevation, with the most significant effect of KAE at 60 mg/kg (ALT, *p* < 0.01; AST, *p* < 0.001) (Figures 2B,C). The Sham and KAE60 groups had normal liver structure and intact liver lobules on morphological and histopathological inspection. In contrast, mice in the HI/RI group showed a larger area of necrosis in the liver, and KAE pretreatment reversed this result (Figure 2D). The area of I/R-induced necrosis was reduced in all KAE pretreatment groups, with the best effect in the KAE60 group. However, the improvement of I/R by KAE 15 mg/kg was less significant (Figure 2D), consistent with serological results (*p* > 0.05) (Figures 2B,C).

### KAE Attenuates I/R-Induced Oxidative Stress *in vivo*

**FIGURE 3:**
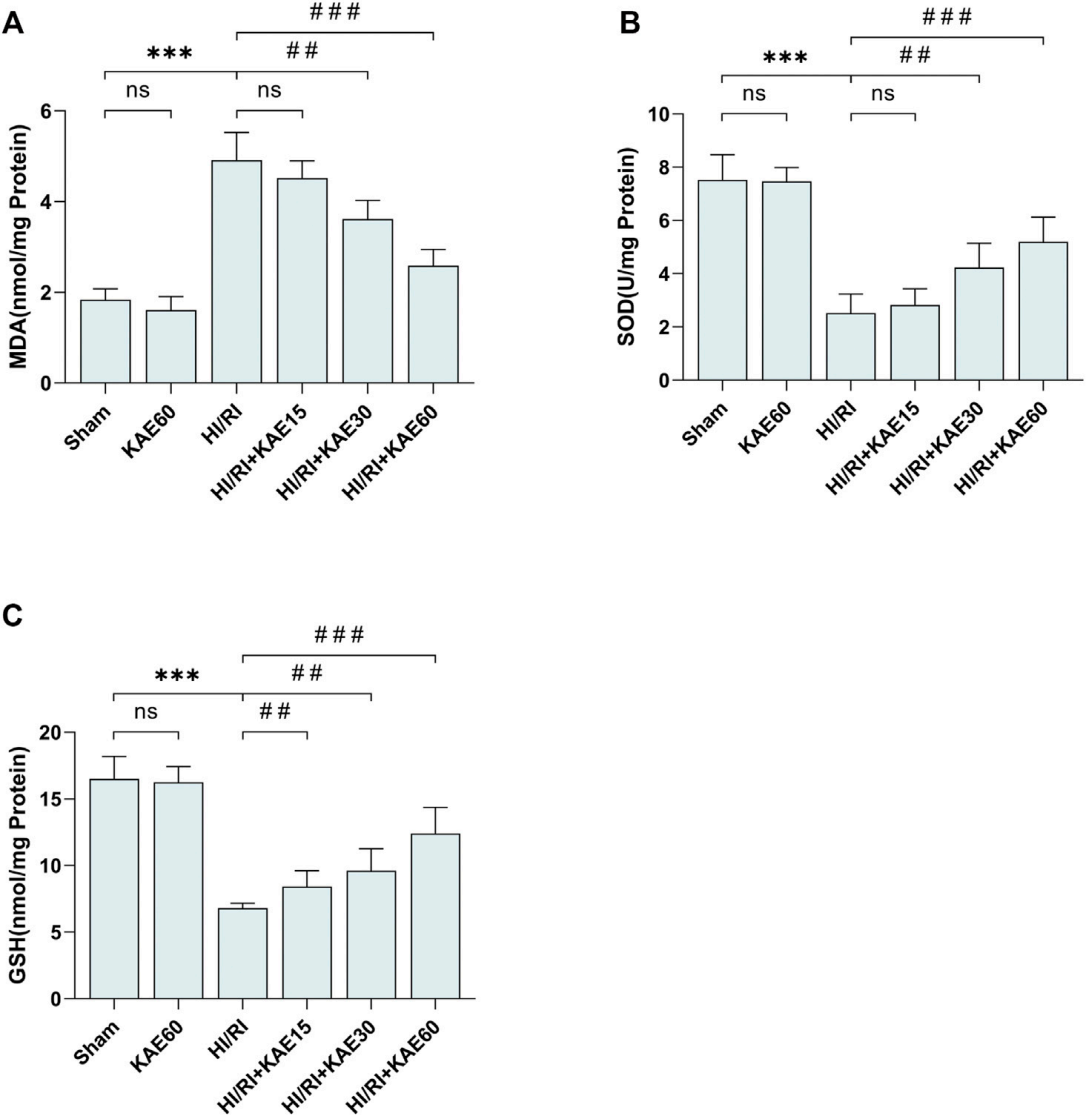
KAE attenuates I/R-induced oxidative stress *in vivo*. Determination of MDA **(A)**, SOD **(B)** and GSH content **(C)** in mice liver tissue homogenates using the corresponding kits. Data were expressed as mean ± standard deviation (SD) values. *p < 0.05, ∗∗*p* < 0.01, and ∗∗∗*p* < 0.001 versus the sham group; ^#^ *p* < 0.05, ^##^ *p* < 0.01, and ^###^ *p* < 0.001 versus the HI/RI group; NS: no significance.

To assess the KAE effect on oxidative stress in the mice HI/RI model, we measured the content of MDA, SOD and GSH in the supernatant of mice liver tissue homogenates as markers of oxidative stress levels (Figures 3A–C). Compared with the Sham group, the MDA content in the liver of mice subjected to I/R injury significantly increased (*p* < 0.001) (Figure 3A), but the KAE pretreatment group was considerably lower than the HI/RI group, especially the HIRI + KAE60 group (*p* < 0.001) (Figure 3A). SOD and GSH, components of the antioxidant system in organisms, were significantly reduced by I/R-induced oxidative stress (*p* < 0.001) (Figures 3B,C), and KAE pretreatment dose-dependently reversed this result, with the best effect at a KAE dose of 60 mg/kg (*p* < 0.001) (Figures 3B,C). However, KAE pretreatment at 15 mg/kg had almost no effect on oxidative stress (Figures 3A–C).

### KAE Suppresses I/R-Induced Inflammation *in vivo*

**FIGURE 4:**
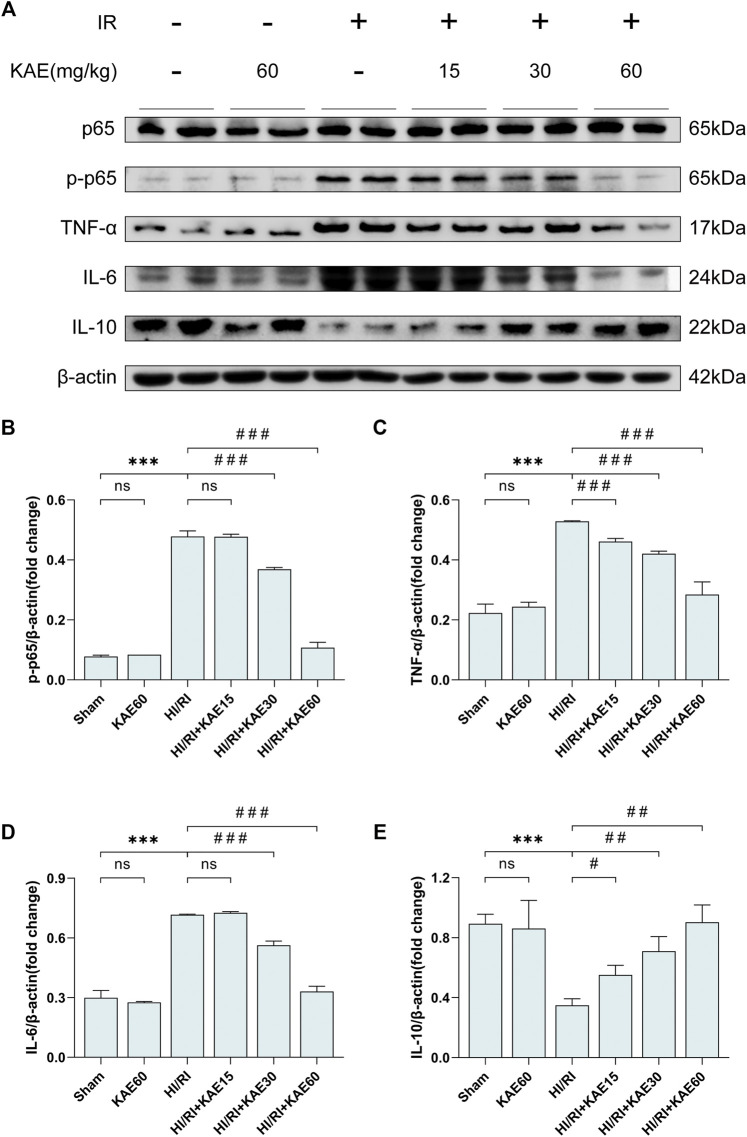
KAE suppresses I/R-induced inflammation *in vivo*. **(A)** Protein expression in liver tissue were determined by western blotting for p65, p-p65, TNF-α, IL-6, IL-10 and β-actin. **(B–E)**Relative protein expression was semi-quantified by analyzing protein grayscale values. Data were expressed as mean ± standard deviation (SD) values. *p < 0.05, ∗∗*p* < 0.01, and ∗∗∗*p* < 0.001 versus the sham group; ^#^ *p* < 0.05, ^##^ *p* < 0.01, and ^###^ *p* < 0.001 versus the HI/RI group; NS: no significance.

To investigate the protective effect of KAE against inflammation induced by I/R, we analyzed the protein expression of inflammatory factors and markers by western blotting. I/R-induced significant NF-κB/p65 phosphorylation and increased the expression of pro-inflammatory factors, including TNF-α and IL-6 (*p* < 0.001) (Figures 4A–D), whereas KAE pretreatment dose-dependently reversed these effects, and the anti-inflammatory effect was most pronounced at a dose of 60 mg/kg (*p* < 0.001) (Figures 4A–D). In addition, the IL-10 involved in anti-inflammatory was significantly downregulated by I/R injury (*p* < 0.001) (Figures 4A,E), whereas KAE pretreatment dose-dependently restores some of the effects (Figures 4A,E). The above results indicate that KAE pretreatment inhibits I/R-induced pro-inflammatory factors release by suppressing NF-κB/p65 activation and increasing the expression of anti-inflammatory factors.

### KAE Alleviates I/R-Induced Hepatocellular Apoptosis *in vivo*

**FIGURE 5:**
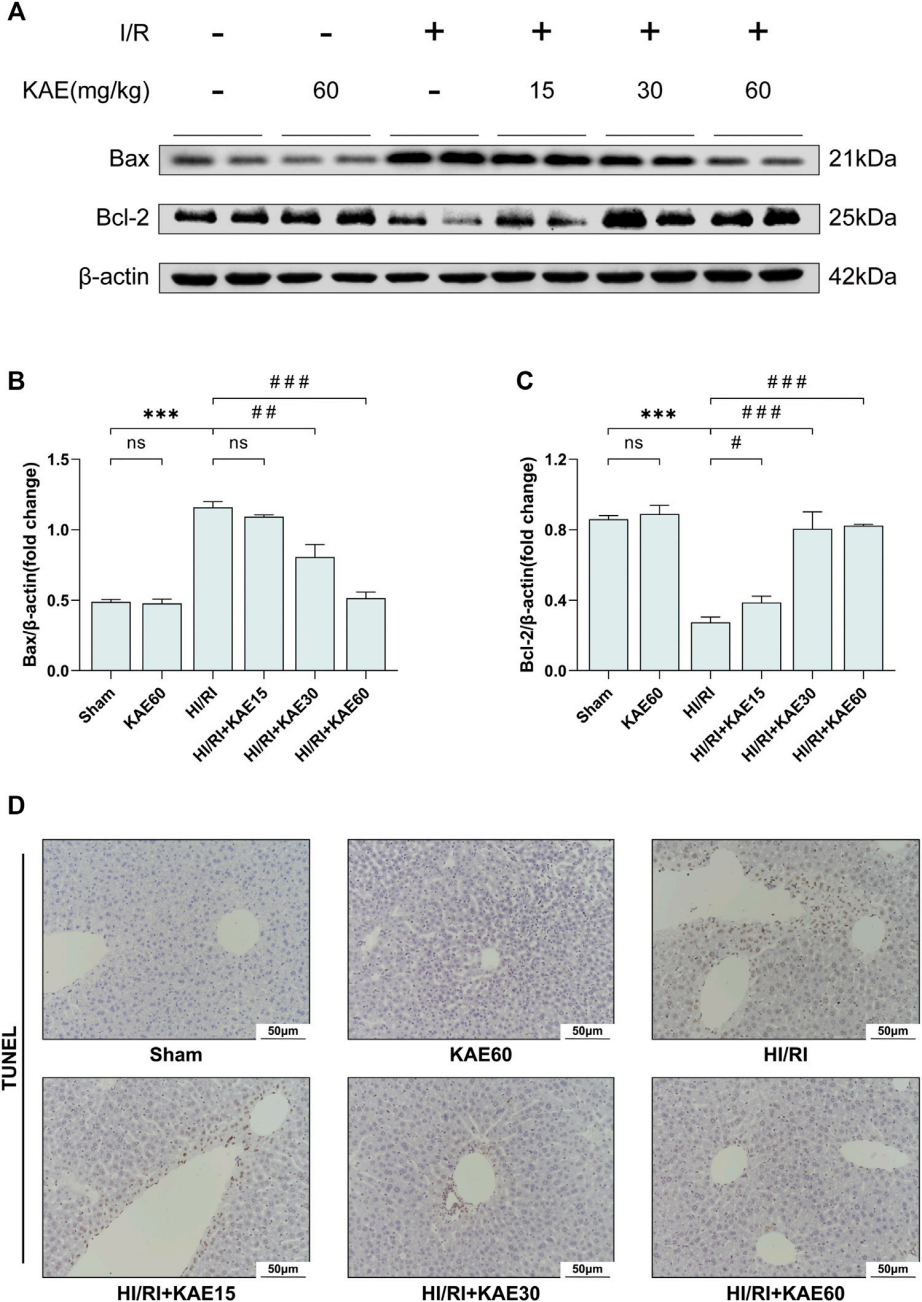
KAE alleviates I/R-induced hepatocellular apoptosis *in vivo*. **(A)** Protein expression in liver tissue were determined by western blotting for Bax, BCL-2, and β-actin; **(B,C)** Relative protein expression was semi-quantified by analyzing protein grayscale values. **(D)** Analysis of hepatocellular apoptosis by TUNEL staining. Data were expressed as mean ± standard deviation (SD) values. *p < 0.05, ∗∗*p* < 0.01, and ∗∗∗*p* < 0.001 versus the sham group; ^#^ *p* < 0.05, ^##^ *p* < 0.01, and ^###^ *p* < 0.001 versus the HI/RI group; NS: no significance.

To further assess the extent of I/R-induced injury *in vivo*, we analyzed the expression of related proteins by western blotting and performed TUNEL staining on liver tissue sections. Western blotting of apoptosis-related protein expression revealed that I/R significantly upregulated the expression of the pro-apoptotic protein Bax while inhibiting the expression of the anti-apoptotic protein Bcl-2 (*p* < 0.001) (Figures 5A–C), which was reversed by KAE pretreatment at an optimal dose of 60 mg/kg (*p* < 0.001) (Figures 5A–C). TUNEL staining was the next section, and our results showed that a large number of TUNEL-positive cells were detected in the liver tissue of the HI/RI group compared with the Sham group (Figure 5D). However, apoptotic hepatocytes in the KAE pretreatment group were significantly lower than those in the HI/RI group, which was particularly significant in the HIRI + KAE60 group (Figure 5D), indicating that KAE pretreatment inhibits I/R-induced hepatocellular apoptosis by downregulating pro-apoptotic protein expression and upregulating anti-apoptotic protein expression.

### KAE Activates the Nrf2/HO-1 Signaling Pathway to Attenuate I/R Injury *in vivo*

**FIGURE 6:**
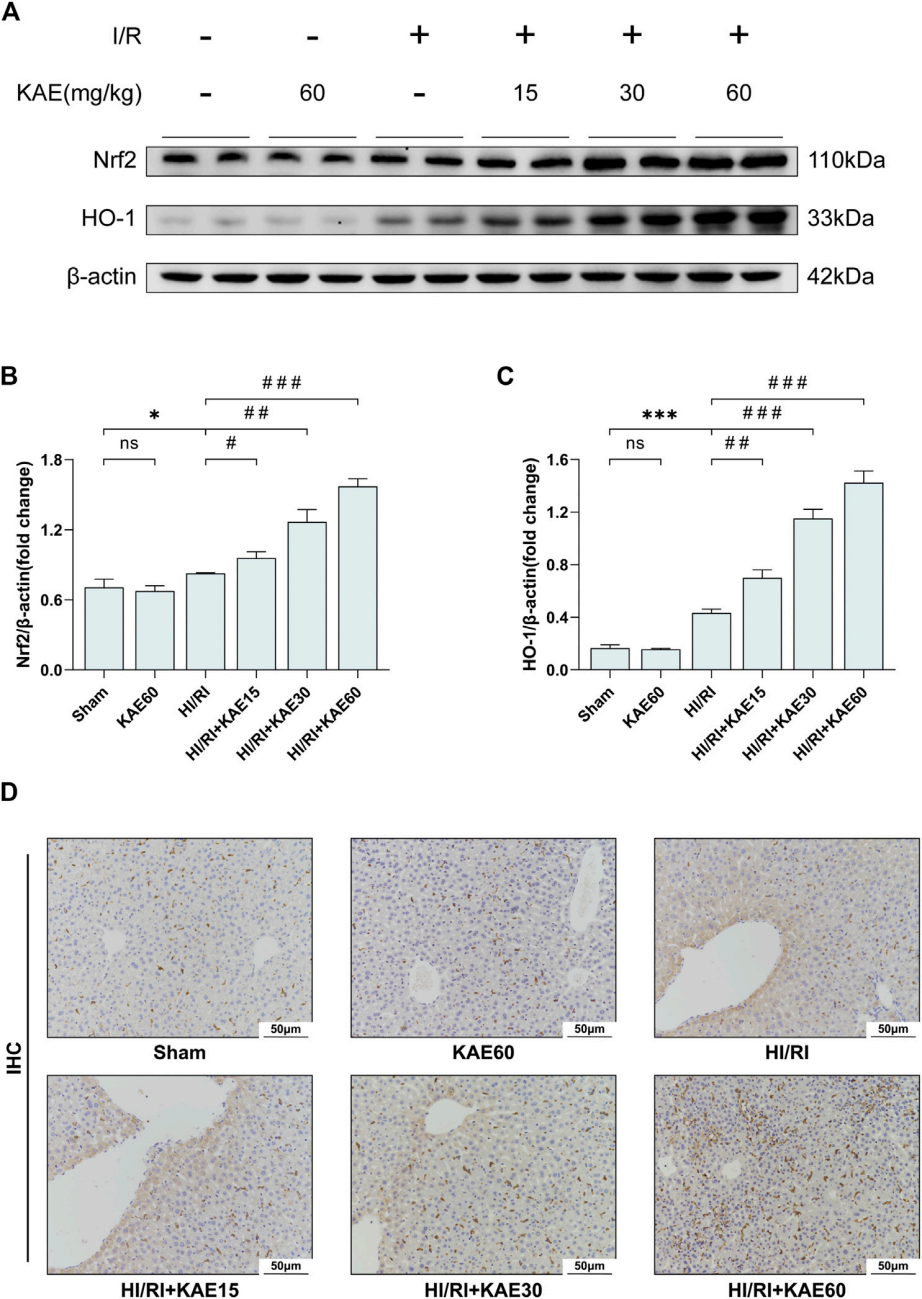
KAE activates the Nrf2/HO-1 signaling pathway to attenuate I/R injury *in vivo*. **(A)** Protein expression in liver tissue were determined by western blotting for Nrf2, HO-1, and β-actin; **(B,C)** Relative protein expression was semi-quantified by analyzing protein grayscale values. **(D)** Analysis of HO-1 expression in mice liver tissues by IHC staining. Data were expressed as mean ± standard deviation (SD) values. *p < 0.05, ∗∗*p* < 0.01, and ∗∗∗*p* < 0.001 versus the sham group; ^#^ *p* < 0.05, ^##^ *p* < 0.01, and ^###^ *p* < 0.001 versus the HI/RI group; NS: no significance.

To investigate the role of KAE in the Nrf2/HO-1 signaling pathway, we analyzed the expression of Nrf2 and HO-1 in total protein extracts from the mice liver by western blotting. Our study showed that I/R injury mildly increased the expression of Nrf2 (*p* < 0.05) (Figures 6A,B) and dramatically increased the expression of HO-1 (*p* < 0.001) (Figures 6A,C) compared with the Sham group. Compared with the HI/RI group, expression of Nrf2 and HO-1 were further increased in the KAE pretreatment groups, and the extent of the increase correlated with the KAE dose, the effect was most pronounced when KAE pretreatment dose reached 60 mg/kg (*p* < 0.001) (Figures 6A,C). IHC staining of HO-1 showed similar results: a minor increase in HO-1-positive cells in the HI/RI group compared with the Sham group, and a significant increase in HO-1-positive cells in the KAE pretreatment group (Figure 6D). These results indicate that Nrf2 was activated under stress conditions and upregulated HO-1 expression to counteract I/R injury, and KAE pretreatment further enhanced the effect.

### KAE Palliates H/R-Induced Hepatocellular Apoptosis *in vitro*

**FIGURE 7:**
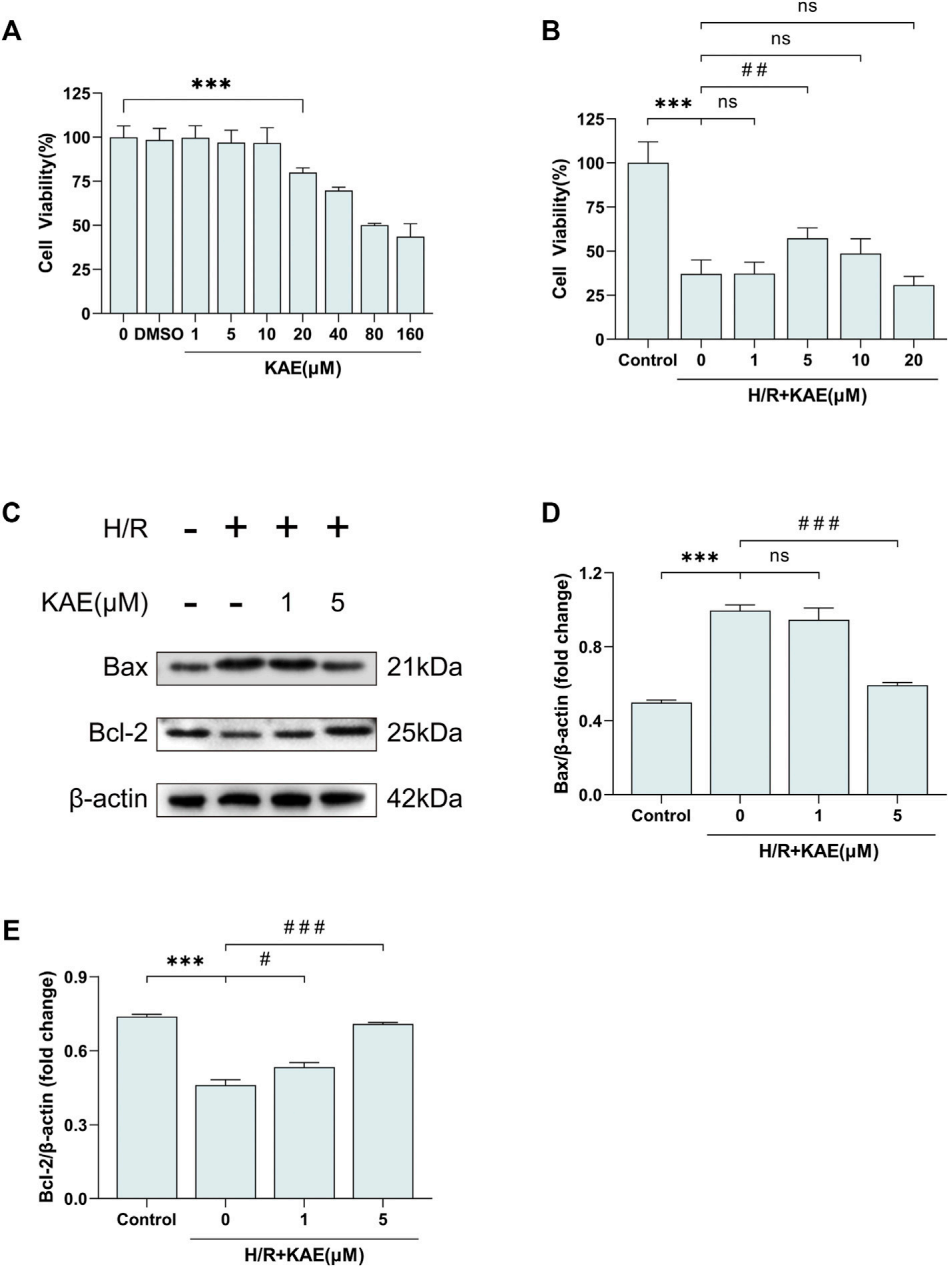
KAE palliates H/R-induced hepatocellular apoptosis *in vitro*. **(A)** Assessment of cytotoxicity of KAE by cell viability assay; **(B)** Screening of optimal concentration of KAE pretreatment by cell viability assay. **(C)** Protein expression in liver tissue was determined by western blotting for Bax, BCL-2, and β-actin; **(D,E)** Relative protein expression was semi-quantified by analyzing protein grayscale values. Data were expressed as mean ± standard deviation (SD) values. *p < 0.05, ∗∗*p* < 0.01, and ∗∗∗*p* < 0.001 versus the control group; ^#^ *p* < 0.05, ^##^ *p* < 0.01, and ^###^ *p* < 0.001 versus the H/R group; NS: no significance.

At the cytological level, we started by pretreating the normal human hepatocyte line QSG-7701 with DMSO and different KAE concentrations for 24 h. Then, cell viability was measured to assess the cytotoxicity of KAE (Figure 7A). Hepatocyte proliferation was significantly inhibited at KAE concentrations up to 20 μM (*p* < 0.001) (Figure 7A), hence KAE concentrations below 20 μM will be used in subsequent experiments. H/R injury significantly inhibited the proliferation of hepatocytes (*p* < 0.001) (Figure 7B), and KAE pretreatment restored the injury, with 5 μM KAE being the optimal concentration (*p* < 0.01) (Figure 7B). Subsequently, western blotting was performed to detect the expression of pro-apoptotic factor Bax and anti-apoptotic factor Bcl-2 in each group of hepatocytes (Figures 7C–E). The results showed that H/R significantly upregulated Bax expression but downregulated the expression of Bcl-2, which was reversed by KAE with an optimal concentration of 5 μM (*p* < 0.001) (Figures 7C–E). These findings are similar to those obtained *in vivo*.

### KAE Reduces ROS Generation and Activates the Nrf2/HO-1 Signaling Pathway to Relieve H/R Injury *in vitro*

**FIGURE 8:**
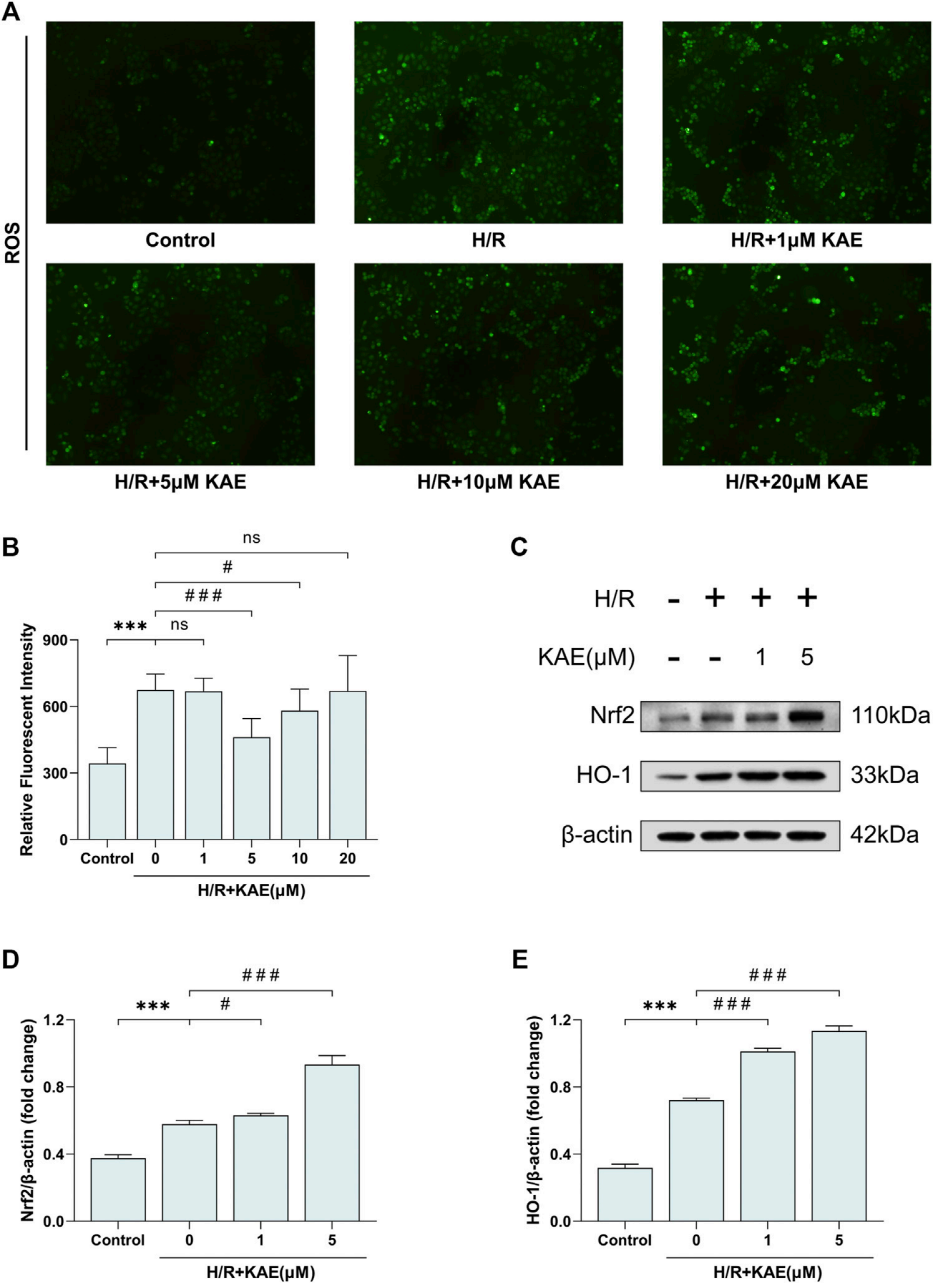
KAE reduces ROS generation and activates the Nrf2/HO-1 signaling pathway to relieve H/R injury *in vitro*. **(A)** Assessment of hepatocellular ROS generation by DCFH-DA fluorescent probe; **(B)** Analysis of ROS relative fluorescence intensity by area scan (3 × 3 reads/well; excitation/emission = 488/525 nm); **(C)** Protein expression in liver tissue were determined by western blotting for Bax, BCL-2, and β-actin; **(D,E)** Relative protein expression was semi-quantified by analyzing protein grayscale values. Data were expressed as mean ± standard deviation (SD) values. *p < 0.05, ∗∗*p* < 0.01, and ∗∗∗*p* < 0.001 versus the control group; ^#^ *p* < 0.05, ^##^ *p* < 0.01, and ^###^ *p* < 0.001 versus the H/R group; NS: no significance.

To assess the level of H/R-induced oxidative stress *in vitro*, the DCFH-DA fluorescent probe was used to label ROS. DCFH-DA fluorescent probe was loaded on hepatocytes, and the fluorescence area was compared to measure ROS levels. Pretreatment with a 5 μM KAE concentration reversed H/R-induced fluorescent area increase, but increasing the KAE concentration to 20 μM may have caused more ROS generation (*p* > 0.05) (Figure 8A). The average fluorescence intensity measurement confirmed the above results (*p* < 0.001) (Figure 8B). Further, we investigated Nrf2 and HO-1 protein expression in hepatocytes under H/R conditions. Compared with the Sham group, H/R injury increased the expression of total Nrf2 and its downstream HO-1 (*p* < 0.001) (Figures 8C–E), which was further enhanced by KAE pretreatment, especially at KAE pretreatment concentration of 5 μM (*p* < 0.001) (Figures 8C–E). The above *in vitro* results were in good agreement with the *in vivo* experiments, confirming the validity of KAE pretreatment.

## Discussion

HI/RI is a common and severe complication in liver surgery, which constrains the development of hepatic surgery, but current therapeutic strategies are limited (Yang et al., 2019). KAE, a flavonoid isolated from *Penthorum chinense* Pursh, has been reported to exhibit significant anti-inflammatory and antioxidant effects (Suchal et al., 2016; Xu et al., 2019; Chen et al., 2020; Du et al., 2020). However, the effects of KAE on HI/RI are yet to be reported. Our study showed that KAE pretreatment significantly reduced I/R-induced impairment of liver function and tissue structure. We attempted to elucidate its possible mechanisms in three dimensions: inflammation, oxidative stress, and apoptosis.

HI/RI manifests as a direct result of hepatocyte injury during the ischemic phase, which induces an inflammatory response, and further cellular dysfunction and injury caused by inflammatory pathway activation (Nace et al., 2013). The anti-inflammatory effect of KAE has been demonstrated in several disease models (Devi et al., 2015), and our study showed that the anti-inflammatory effect of KAE is closely related to the inhibition of NF-κB phosphorylation. NF-κB/p65, as one of the primary regulators of classical inflammatory pathways, plays a vital role in the occurrence and progression of ischemia-reperfusion injury in multiple organs (Zhang et al., 2020). In addition, our study indicated that KAE downregulated the expression of pro-inflammatory factors (including TNF-α and IL-6) by inhibiting the activation of NF-κB/p65.

When it comes to oxidative stress, as mentioned previously, the occurrence and progression of HI/RI are closely related to oxidative stress, and ROS is a key link in it (Elias-Miró et al., 2013). Therefore, we measured the content of MDA, SOD, and GSH *in vivo* and the level of ROS *in vitro* to represent the degree of oxidative stress. Several existing studies have demonstrated the potential of KAE on scavenging ROS and mitigating oxidative stress (Saw et al., 2014; Zeka et al., 2020). In the present study, our data confirmed that KAE exerts antioxidant effects by activating the Nrf2/HO-1 signaling pathway.

Further investigation of the hepatoprotective effects of KAE revealed that KAE pretreatment attenuates inflammation and oxidative stress under stressful conditions *in vivo* and *in vitro* and remarkably alleviates hepatocyte apoptosis. It has long been demonstrated that Bcl-2 is a critical anti-apoptotic protein in organisms and promoting Bcl-2 expression significantly alleviates HI/RI (Selzner et al., 2002). Similarly, our results suggested that KAE downregulates the expression of apoptotic protein Bax and upregulates the expression of anti-apoptotic protein Bcl-2 in I/R and H/R-induced injury, thereby alleviating hepatocyte apoptosis. Also, TUNEL staining of liver sections supported the results of western blotting.

Our study on the mechanism of KAE revealed that the potent anti-inflammatory, antioxidant and anti-apoptotic effects of KAE might be attributed to the activation of the Nrf2/HO-1 signaling pathway by KAE. Previous studies have demonstrated that the Nrf2/HO-1 signaling pathway is one of the critical pathways for biological resistance to inflammation and oxidative stress, and the transcriptional response of Nrf2 is essential for maintaining homeostasis in the organism (Bardallo et al., 2021). Under physiological conditions, Nrf2 is anchored in the cytoplasm by binding to its inhibitor, Kelch-like ECH-associated protein-1 (Keap1). Various endogenous or exogenous stimuli dissociate Nrf2 from Keap1, resulting in nuclear translocation of Nrf2, which in turn mediates transcriptional activation of antioxidant response element (ARE) regulatory genes, thereby reducing ROS levels, inflammation, and cell death (Bataille and Manautou, 2012; Galicia-Moreno et al., 2020; Jayasuriya et al., 2021). Among the genes downstream of ARE-mediated transcriptional activation, up-regulation of HO-1 may be one of the most critical cytoprotective mechanisms activated during cellular stress, such as inflammation, ischemia, hypoxia, hyperoxia, hyperthermia, or radiation (Brockmann et al., 2005). Moreover, HO-1 was thought to play a crucial role in maintaining antioxidant/oxidant balance during cellular injury (Waltz et al., 2011), and its anti-inflammatory and anti-apoptotic effects have been validated in multiple disease models (McDaid et al., 2005).

Notably, studies showed that the expression of Nrf2 and HO-1 were downregulated under stress conditions (Yao et al., 2020; Yu et al., 2021). In addition, KAE was found to upregulate the expression of Nrf2 and HO-1 in WT mice and cells without any treatment (Yao et al., 2020). However, in contrast to earlier findings, our results demonstrate that KAE pretreatment has no significant effect under physiological conditions (Sham group versus KAE60 group *in vivo*). Meanwhile, Nrf2 and HO-1 expression was mildly elevated under stress conditions (Sham group versus HI/RI group *in vivo*; Control group versus H/R group *in vitro*), and the effect was dose-dependently enhanced by KAE pretreatment. Our results are similar to some studies that indicated that upregulation of Nrf2 and HO-1 expression was associated with anti-inflammatory and antioxidant functions initiated by hepatocytes during HI/RI (Li et al., 2021; Ma et al., 2021; Zhuang et al., 2021). Collectively, our results suggested that I/R injury upregulated Nrf2 and HO-1 to some extent to counteract stress and injury and that KAE pretreatment has a significant effect only when mice are subjected to I/R injury.

## Conclusion

This study verified the protective effects of KAE pretreatment on the liver from both animal and cellular perspectives. Its potent anti-inflammatory and antioxidant effects were associated with inhibition of the NF-κB/p65 and activation of the Nrf2/HO-1 signaling pathway. Based on the existing studies, the current study further elaborated the specific mechanism of KAE to alleviate HI/RI. These results suggest promising drug candidates for preventing and treating HI/RI and laying the foundation for the development and application of *Penthorum chinense* Pursh.

## Data Availability

The original contributions presented in the study are included in the article/Supplementary Material, further inquiries can be directed to the corresponding authors.

## References

[B1] Abu-Amara M. Yang S. Y. Tapuria N. Fuller B. Davidson B. Seifalian A. (2010). Liver Ischemia/reperfusion Injury: Processes in Inflammatory Networks-Aa Review. Liver Transpl. 16, 1016–1032. 10.1002/lt.22117 20818739

[B9] Bardallo R. G. Panisello‐Roselló A. Sanchez‐Nuno S. Alva N. Roselló‐Catafau J. Carbonell T. (2021). Nrf2 and Oxidative Stress in Liver Ischemia/reperfusion Injury. FEBS J. [Epub ahead of print]. 10.1111/febs.16336 34967991

[B2] Bataille A. M. Manautou J. E. (2012). Nrf2: a Potential Target for New Therapeutics in Liver Disease. Clin. Pharmacol. Ther. 92, 340–348. 10.1038/clpt.2012.110 22871994 PMC3704160

[B3] Brockmann J. G. August C. Wolters H. H. Hömme R. Palmes D. Baba H. (2005). Sequence of Reperfusion Influences Ischemia/reperfusion Injury and Primary Graft Function Following Porcine Liver Transplantation. Liver Transpl. 11, 1214–1222. 10.1002/lt.20480 16184569

[B4] Chen J. Xuan Y. H. Luo M. X. Ni X. G. Ling L. Q. Hu S. J. (2020). Kaempferol Alleviates Acute Alcoholic Liver Injury in Mice by Regulating Intestinal Tight junction Proteins and Butyrate Receptors and Transporters. Toxicology 429, 152338. 10.1016/j.tox.2019.152338 31785310

[B5] Devi K. P. Malar D. S. Nabavi S. F. Sureda A. Xiao J. Nabavi S. M. (2015). Kaempferol and Inflammation: From Chemistry to Medicine. Pharmacol. Res. 99, 1–10. 10.1016/j.phrs.2015.05.002 25982933

[B6] Du Y. C. Lai L. Zhang H. Zhong F. R. Cheng H. L. Qian B. L. (2020). Kaempferol from Penthorum Chinense Pursh Suppresses HMGB1/TLR4/NF-Κb Signaling and NLRP3 Inflammasome Activation in Acetaminophen-Induced Hepatotoxicity. Food Funct. 11, 7925–7934. 10.1039/d0fo00724b 32820776

[B7] Elias-Miró M. Jiménez-Castro M. B. Rodés J. Peralta C. (2013). Current Knowledge on Oxidative Stress in Hepatic Ischemia/reperfusion. Free Radic. Res. 47, 555–568. 10.3109/10715762.2013.811721 23738581

[B8] Eltzschig H. K. Eckle T. (2011). Ischemia and Reperfusion-Ffrom Mechanism to Translation. Nat. Med. 17, 1391–1401. 10.1038/nm.2507 22064429 PMC3886192

[B10] Galicia-Moreno M. Lucano-Landeros S. Monroy-Ramirez H. C. Silva-Gomez J. Gutierrez-Cuevas J. Santos A. (2020). Roles of Nrf2 in Liver Diseases: Molecular, Pharmacological, and Epigenetic Aspects. Antioxidants 9, 980. 10.3390/antiox9100980 PMC7601324 33066023

[B11] Guo W. Jiang Y. Chen X. Yu P. Wang M. Wu X. (2015). Identification and Quantitation of Major Phenolic Compounds from Penthorum Chinense Pursh. By HPLC with Tandem Mass Spectrometry and HPLC with Diode Array Detection. J. Sep. Sci. 38, 2789–2796. 10.1002/jssc.201500303 26037645

[B12] Jayasuriya R. Dhamodharan U. Ali D. Ganesan K. Xu B. Ramkumar K. M. (2021). Targeting Nrf2/Keap1 Signaling Pathway by Bioactive Natural Agents: Possible Therapeutic Strategy to Combat Liver Disease. Phytomedicine 92, 153755. 10.1016/j.phymed.2021.153755 34583226

[B13] Jochmans I. Meurisse N. Neyrinck A. Verhaegen M. Monbaliu D. Pirenne J. (2017). Hepatic Ischemia/reperfusion Injury Associates with Acute Kidney Injury in Liver Transplantation: Prospective Cohort Study. Liver Transpl. 23, 634–644. 10.1002/lt.24728 28124458

[B14] Ju C. Tacke F. (2016). Hepatic Macrophages in Homeostasis and Liver Diseases: from Pathogenesis to Novel Therapeutic Strategies. Cell Mol Immunol 13, 316–327. 10.1038/cmi.2015.104 26908374 PMC4856798

[B15] Lei X. F. Fu W. Kim-Kaneyama J. R. Omoto T. Miyazaki T. Li B. (2016). Hic-5 Deficiency Attenuates the Activation of Hepatic Stellate Cells and Liver Fibrosis through Upregulation of Smad7 in Mice. J. Hepatol. 64, 110–117. 10.1016/j.jhep.2015.08.026 26334580

[B16] Li Z. Wang Y. Zhang Y. Wang X. Gao B. Li Y. (2021). Protective Effects of Fisetin on Hepatic Ischemia-Reperfusion Injury through Alleviation of Apoptosis and Oxidative Stress. Arch. Med. Res. 52, 163–173. 10.1016/j.arcmed.2020.10.009 33645502

[B17] Ma H. Yang B. Yu L. Gao Y. Ye X. Liu Y. (2021). Sevoflurane Protects the Liver from Ischemia-Reperfusion Injury by Regulating Nrf2/HO-1 Pathway. Eur. J. Pharmacol. 898, 173932. 10.1016/j.ejphar.2021.173932 33631180

[B18] Mcdaid J. Yamashita K. Chora A. Ollinger R. Strom T. B. Li X. C. (2005). Heme Oxygenase-1 Modulates the Allo-Immune Response by Promoting Activation-Induced Cell Death of T Cells. Faseb j 19, 458–460. 10.1096/fj.04-2217fje 15640283

[B19] Nace G. W. Huang H. Klune J. R. Eid R. E. Rosborough B. R. Korff S. (2013). Cellular-specific Role of Toll-like Receptor 4 in Hepatic Ischemia-Reperfusion Injury in Mice. Hepatology 58, 374–387. 10.1002/hep.26346 23460269 PMC3688695

[B20] Pizzino G. Irrera N. Cucinotta M. Pallio G. Mannino F. Arcoraci V. (2017). Oxidative Stress: Harms and Benefits for Human Health, Oxidative Med. Cell. longevity 2017, 1–13. 10.1155/2017/8416763 PMC5551541 28819546

[B21] Rabha D. J. Singh T. U. Rungsung S. Kumar T. Parida S. Lingaraju M. C. (2018). Kaempferol Attenuates Acute Lung Injury in Caecal Ligation and Puncture Model of Sepsis in Mice. Exp. Lung Res. 44, 63–78. 10.1080/01902148.2017.1420271 29393707

[B22] Saw C. L. Guo Y. Yang A. Y. Paredes-Gonzalez X. Ramirez C. Pung D. (2014). The berry Constituents Quercetin, Kaempferol, and Pterostilbene Synergistically Attenuate Reactive Oxygen Species: Involvement of the Nrf2-ARE Signaling Pathway. Food Chem. Toxicol. 72, 303–311. 10.1016/j.fct.2014.07.038 25111660

[B23] Selzner M. Rüdiger H. A. Selzner N. Thomas D. W. Sindram D. Clavien P. A. (2002). Transgenic Mice Overexpressing Human Bcl-2 Are Resistant to Hepatic Ischemia and Reperfusion. J. Hepatol. 36, 218–225. 10.1016/s0168-8278(01)00259-810.1016/s0168-8278(01)00259-8 11830333

[B24] Subramanya S. B. Venkataraman B. Meeran M. F. N. Goyal S. N. Patil C. R. Ojha S. (2018). Therapeutic Potential of Plants and Plant Derived Phytochemicals against Acetaminophen-Induced Liver Injury. Int. J. Mol. Sci. 19, 3776. 10.3390/ijms19123776 PMC6321362 30486484

[B25] Suchal K. Malik S. Gamad N. Malhotra R. K. Goyal S. N. Chaudhary U. (20162016). Kaempferol Attenuates Myocardial Ischemic Injury via Inhibition of MAPK Signaling Pathway in Experimental Model of Myocardial Ischemia-Reperfusion Injury. Oxid Med. Cel Longev 2016, 7580731. 10.1155/2016/7580731 PMC4819110 27087891

[B26] Waltz P. Carchman E. H. Young A. C. Rao J. Rosengart M. R. Kaczorowski D. (2011). Lipopolysaccaride Induces Autophagic Signaling in Macrophages via a TLR4, Heme Oxygenase-1 Dependent Pathway. Autophagy 7, 315–320. 10.4161/auto.7.3.14044 21307647

[B27] Wang A. Lin L. Wang Y. (2015). Traditional Chinese Herbal Medicine Penthorum Chinense Pursh: A Phytochemical and Pharmacological Review. Am. J. Chin. Med. 43, 601–620. 10.1142/s0192415x15500378 26119956

[B28] Xu T. Huang S. Huang Q. Ming Z. Wang M. Li R. (2019). Kaempferol Attenuates Liver Fibrosis by Inhibiting Activin Receptor-like Kinase 5. J. Cel Mol Med 23, 6403–6410. 10.1111/jcmm.14528 PMC6714241 31273920

[B29] Yang L. Wang W. Wang X. Zhao J. Xiao L. Gui W. (2019). Creg in Hepatocytes Ameliorates Liver Ischemia/Reperfusion Injury in a TAK1-dependent Manner in Mice. Hepatology 69, 294–313. 10.1002/hep.30203 30076625

[B30] Yao H. Sun J. Wei J. Zhang X. Chen B. Lin Y. (2020). Kaempferol Protects Blood Vessels from Damage Induced by Oxidative Stress and Inflammation in Association with the Nrf2/HO-1 Signaling Pathway. Front. Pharmacol. 11, 1118. 10.3389/fphar.2020.01118 32792954 PMC7387620

[B31] Yu Q. Chen S. Tang H. Zhang X. Tao R. Yan Z. (2021). Veratric Acid Alleviates Liver Ischemia/reperfusion Injury by Activating the Nrf2 Signaling Pathway. Int. Immunopharmacol 101, 108294. 10.1016/j.intimp.2021.108294 34749250

[B32] Zeka K. Marrazzo P. Micucci M. Ruparelia K. C. Arroo R. R. J. Macchiarelli G. (2020). Activity of Antioxidants from Crocus Sativus L. Petals: Potential Preventive Effects towards Cardiovascular System. Antioxidants (Basel) 9. 10.3390/antiox9111102 PMC7697793 33182461

[B33] Zhang S. Feng Z. Gao W. Duan Y. Fan G. Geng X. (2020). Aucubin Attenuates Liver Ischemia-Reperfusion Injury by Inhibiting the HMGB1/TLR-4/nf-Κb Signaling Pathway, Oxidative Stress, and Apoptosis. Front. Pharmacol. 11, 544124. 10.3389/fphar.2020.544124 33013386 PMC7506056

[B34] Zhuang L. Ding W. Zhang Q. Ding W. Xu X. Yu X. (2021). TGR5 Attenuated Liver Ischemia-Reperfusion Injury by Activating the Keap1-Nrf2 Signaling Pathway in Mice. Inflammation 44, 859–872. 10.1007/s10753-020-01382-y 33169298

